# Animal Electricity

**Published:** 1867-09

**Authors:** 


(To be Continued.)

---

### ARTICLE II.

### *Animal Electricity.*

Popular ignorance is the fruitful soil in which all manner of humbugs germinate speedily, grow rapidly and fructify abundantly. Yet it is not absolute ignorance which furnishes so kindly a soil to mushroom theories and fungous sham science, but rather that half-knowledge, in which native stupidity has been fertilized by the offal of science, rejected by its true votaries and descending, like the cast off clothes of beaux and belles, through various grades of society, to its ultimate destination, the rubbish-heap.

Nowhere has this half-learning, which is really the worst ignorance, revelled to such an extent as in the department of electricity. Every unaccountable phenomena is supposed to be explained by the simple utterance of the talismanic word, electricity. Does a table tip, or a heavy piano walk up stairs at the bidding of a medium, electricity is supposed to be the motive power. This physical force is to the sciolists of our day, what magic was to the more honest ignoramuses of the past. The less they know of it, the more willing they are to invoke its aid, and the more tremendous are the occult powers they attribute to it.

It is a very comical physiology which these electromaniacs have rendered popular among the so-called educated people of this continent. It is no easy matter to reduce these ravings to definite form, and we shall not attempt it. All our readers, however, must have heard statements from peripatetic lecturers that the brain was the positive and the stomach the negative pole of a great galvanic battery, and that by the electrical reactions of these, nervous fluid or life was generated. Electroquacks professed to heal all the ills of mortal life by cunningly devised currents of electricity, to drive disease from point to point till it was ultimately hounded out of the system; and a generation which prides itself upon its enlightenment, actually believed all this unmitigated nonsense.

Animal electricity has also been misunderstood by men who are greatly superior to these quacks and their gulls. When Galvani had published an account of his famous experiment on frogs, the scientific world was greatly excited at the new discovery. It was fondly imagined that electrified plants grew with unwonted vigour, that the soil could be fertilized by electricity, that an amalgam of zinc could perform the normal functions of an animal's brain. The electrical phenomena produced by rubbing with the hand the back of a living cat were supposed to demonstrate the existence of vital electricity, whereas in truth the very same manifestations are obtained when a dry warm cat-skin is substituted for the living animal. The currents, which the galvanometer shows to exist when one end of a wire touches the interior of the mouth, and the other the skin of the face moistened with perspiration, were explained in the same way; but we know that sweat is acid and saliva alkaline, and that a current always takes place from an alkaline to an acid fluid when the proper connexions are made. The thickening of the white of an egg along the course of an electric current was supposed to be the commencement of organization, but in reality, the albumen was simply cooked hard by the heat of the current, as it would have been by that of an ordinary fire.

In a similar manner may we account for those higher manifestations of a supposed connection between electricity and organization. Thus the peculiar circulation of the chara, an aquatic plant, is stopped by the electric current, but who knows what coagulation of the juices it may have occasioned? Seeds, frequently exposed in the focus of an ordinary electric machine, germinate more quickly than others, but in that focus *ozone* is always generated, and ozone notoriously promotes germination. So too, upon a piece of cloth moistened with a saline solution, small seeds exposed to an electric current have been found to germinate more rapidly at the negative than at the positive electrode. Now this is only an indirect action of the electric force. The current decomposes the saline solution, developing the alkali at the negative and the acid at the positive electrode, and alkalies favor germination, while acids retard it.

In considering real electro physiological phenomena, the *electric shock*, that is, the pain and involuntary muscular contraction which occur when an electric current is passed through a living person, naturally presents itself, as one of the most familiar of these manifestations. Now contractility is an inherent muscular property, the immediate cause of which we do not now propose to consider. A striking proof of this fact is an experiment of Matteuci, to whose lectures in Turin we once for all acknowledge our indebtedness for the facts and most of the principles of this article. He took two frogs one of which was killed by mechanical violence, the other poisoned by *curare*. A current sent through the nerves of the former, produced violent contraction, but when passed through those of the poisoned frog, no contraction took place. The muscles, however, instantly contracted when the current was passed through them.

One of the marked phenomena of the shock is that the pain is chiefly felt at the articulations. This is due to the conditions of electrical transmission. Moist skin receives and conveys the shock more rapidly than dry; muscle, being full of blood and saline solutions, transmits electricity five or six times better than nerve. On this account the section of the conductor is greatly narrowed at the joints where the muscular mass is smaller or nearly absent, and the electric tension in the nerves at those points is much greater than where they are imbedded in a large collection of muscular fibres.

The rapidity and force of muscular contraction have been carefully measured. It appears as the average of numerous observations, that at the beginning of the experiments on recently killed frogs, while the vitality of the muscle is still considerable, the duration of the contraction is 1.100th of a second; the entire contraction, (i. e. the contraction and relaxation) occupies $\frac{1}{4}$ to $\frac{1}{3}$ of a second. A very strong electrical discharge will keep up the contraction longer, and sometimes render it permanent.

In considering these phenomena, it is necessary to bear in mind that physical and chemical phenomena are not brought about under the same conditions as vital manifestations. The quantity of water decomposed, for example, or the amount of heat generated by an electrical current depends upon the quantity of electricity passing in a unit of time. The physiological phenomena of contraction and pain, however, occur only at the beginning and ending of the passage of the current. It is not therefore the quantity of electricity which produces these phenomena, but the variation of the electric condition of the nerves and muscles at the moment of opening or closing a voltaic circuit. By a series of repeated shocks, it is possible to exhaust nervous power and kill the animal, though the current itself may be quite weak.

So susceptible are the nerves and muscles to this change of electric condition, that they are far more delicate indicator of the presence of electricity, than the most accurate electrometer. A Leyden jar discharged repeatedly by a metallic arc, and which fails to effect the nicest electroscope, will nevertheless excite contractions in the muscles of a frog. The quantity of zinc consumed in the battery, bears no proportion to the amount of muscular contraction induced by it. In all merely chemical and physical phenomena of electricity, there is a simple and definite relation between the result obtained and the amount of zinc consumed in the battery. And this relation is expressed by the equivalent of the heat developed during the consumption of the zinc. Now when we compute the mechanical effect of muscular contraction induced by a galvanic current, we find that it is some thirty thousand times greater than this heat-equivalent. Evidently then, there is a new force developed, just as the spark applied to gunpowder produces an effect far beyond any power residing in itself, by inducing a new combination of elements already existing in a quiescent condition.

The evidence of the independence and muscular origin of this contraction may be gathered from the following considerations. An experiment with dead frogs suspended from wires in bottles, shows that the exhalation of carbonic acid is very much greater from those in which muscular contractions have been induced, than from those which have been suffered to remain at rest. Muscles which have contracted repeatedly, give off quantities of carbonic acid, and Bernard discovered that the blood coming from contracted muscles contains no oxygen, but much carbonic acid. It would be erroneous to suppose that this carbonic acid is the primary product of muscular contraction. There are, on the contrary, fixed acids developed by this act.

The mechanical force of contraction is not the only one developed by the chemical changes which have been thus briefly glanced at. Heat is also produced, as is easily made manifest by Melloni's thermo-electric apparatus.

From these considerations it would appear that the contraction is not the direct result of the electric shock, but that the current first induces chemical changes, and that these chemical changes bring about the shortening of the fibre. This shortening has been compared to the approximation of the rings of a soft iron spiral, suspended in a helix, when the current passes. This suffices for an illustration, but is too crude for an explanation. The approximation of the muscular discs is more complex than a mere magnetic polarity.

